# Supplementary material for: Physical Activity Detection for Diabetes Mellitus Patients Using Recurrent Neural Networks
Source: Sensors (Basel). 2024 Apr 10;24(8):2412. doi: 10.3390/s24082412 (PMC11054023; doi:10.3390/s24082412)
Supplement: Supplementary file 1 [file sensors-24-02412-s001.zip › sensors-2910621-supplementary.pdf]

Besides F1-score presented in the main text, it is analyzed which parameter configuration is already sufficient to achieve the required performance. The used metrics are Accuracy, Precision, Recall. These metrics are numerically examined for the top 30 models for both GRU and LSTM and visualized using box plots. This section complements the F1 score results already presented in the Results section.

1. Precision

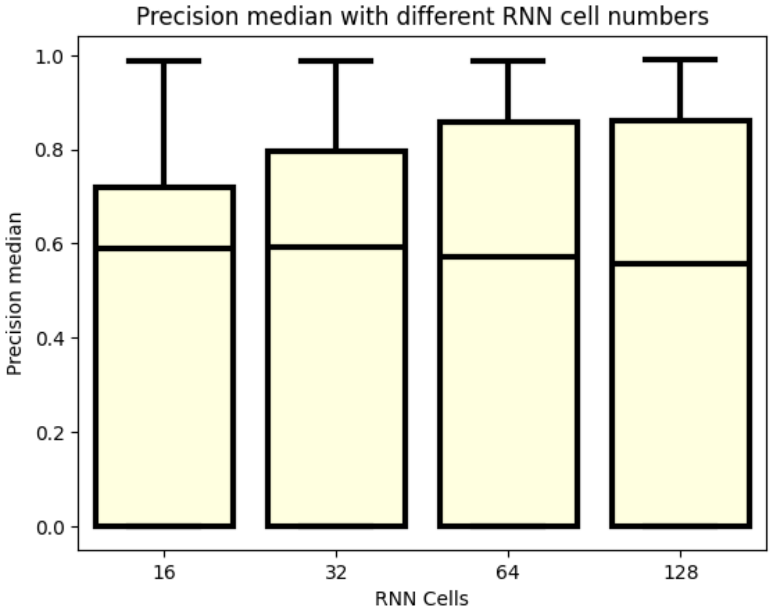

Figure S1. Precision value for different number off RNN cell

Let's begin by analyzing the boxplot results for Figure S1 the precision values corresponding to different sizes of RNN cells (16, 32, 64, 128). The median precision values for each cell size are calculated and grouped based on other variables. One notable observation is that all four configurations exhibit instances of high performance based on precision metrics, with maximum values approaching unity. However, it is noteworthy that the median precision value tends to decrease as the number of RNN cells increases. This phenomenon can be attributed to the issue of gradient vanishing, whereby larger recurrent stack sizes lead to a greater loss of information. Nevertheless, models with larger numbers of recurrent cells generally outperform those with only 16 cells. Additionally, the upper quartile values are higher for configurations utilizing 32, 64, and 128 cells. However, for configurations with 64 and 128 cells, the performance begins to plateau. Consequently, it can be inferred that, from a precision standpoint, employing 64 recurrent layers is adequate for achieving good performance. Furthermore, utilizing a higher number of cells entails longer learning times. Nonetheless, overall, the number of RNN cells does not significantly impact the model's performance.

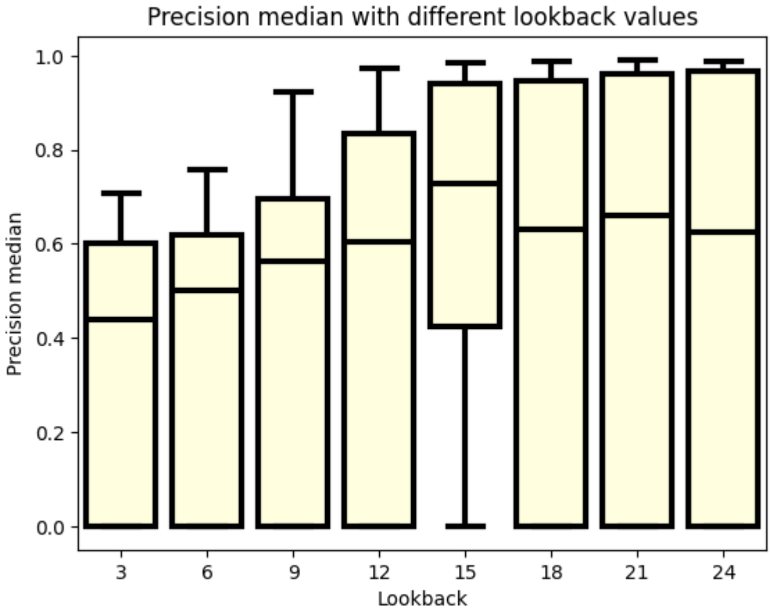

Figure S2. Precision value for different number off Look back

Let's analyze how the look-back window impacts the Precision metric, as depicted in Figure S2. The median Precision value is computed for each look-back value, ranging from 3 to 24. These values correspond to data observations taken at intervals of 5 minutes, resulting in a look-back window spanning from a quarter-hour to two hours, with a quarter-hour difference between each value. Upon examination of the boxplot, a noticeable staircase pattern emerges, with the median values gradually increasing from a look-back value of 3 to 15. This trend is similarly observed for the upper quartile and maximum values. However, beyond the 15th value, there is no further improvement in results. Although the upper quartile values continue to increase, the median values begin to deteriorate, while the maximum values plateau. This suggests that a look-back value of 15 is sufficient for achieving a well-performing system. Nonetheless, employing the maximum look-back value of 24 may yield slightly improved results, albeit with increased learning time.

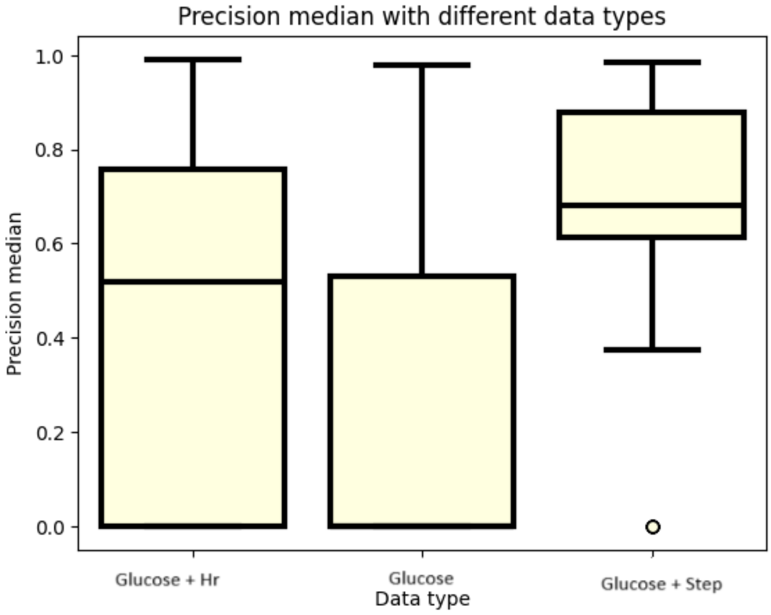

Figure S3. Precision value for different datatype

Figure S3 presents boxplots comparing the performance metrics based on the type of data utilized. Three scenarios are considered: using only blood glucose level data, using blood glucose level and heart rate data, and using blood glucose level and step count data. The analysis focuses on the median Precision values. Upon initial inspection, it becomes evident that relying solely on blood glucose level data is insufficient. This inadequacy stems from the delayed response of the model to changes in blood glucose levels, as these changes are reflected with a lag. However, incorporating step count or heart rate data aids in processing sudden changes until adjustments in blood glucose levels occur. Notably, utilizing step count data yields superior performance compared to heart rate data. An interesting observation is that the minimum Precision value is not zero when heart rate data is included. Overall, models perform best when incorporating step count data alongside blood glucose level data.

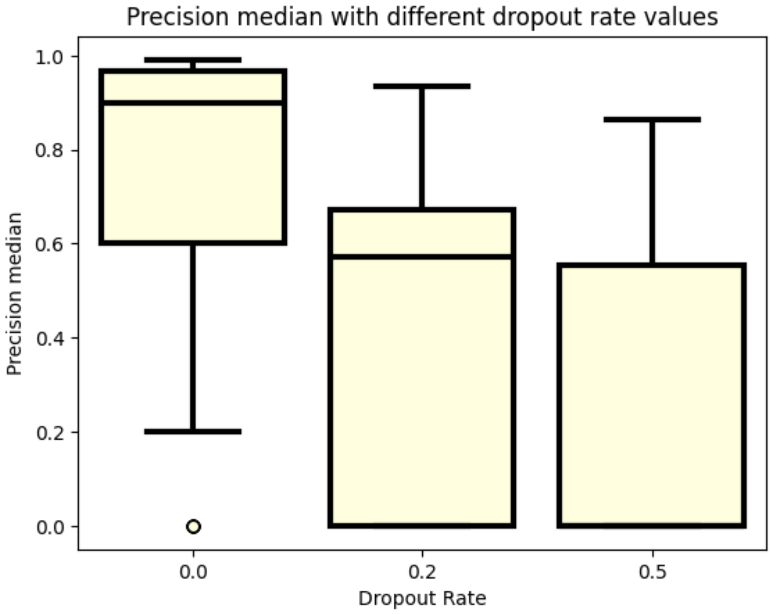

Figure S4. Precision value for different number off Drop out rate

Figure S4 the examination of different dropout rates, with values of 0.0, 0.2, and 0.5. Dropout rates are implemented to prevent overfitting; however, excessively high dropout rates can impede the model's ability to learn from the dataset effectively.In this analysis, it is observed that employing a dropout rate of 0.0, indicating no dropout, allows the model to achieve notably high performance. Conversely, the introduction of dropout rates leads to a deterioration in model performance. Specifically, as dropout rates increase, the lower quartile values approach zero, indicating poorer performance. Additionally, for a dropout rate of 0.5, the median value is also close to zero, further suggesting diminished model effectiveness under higher dropout rates.

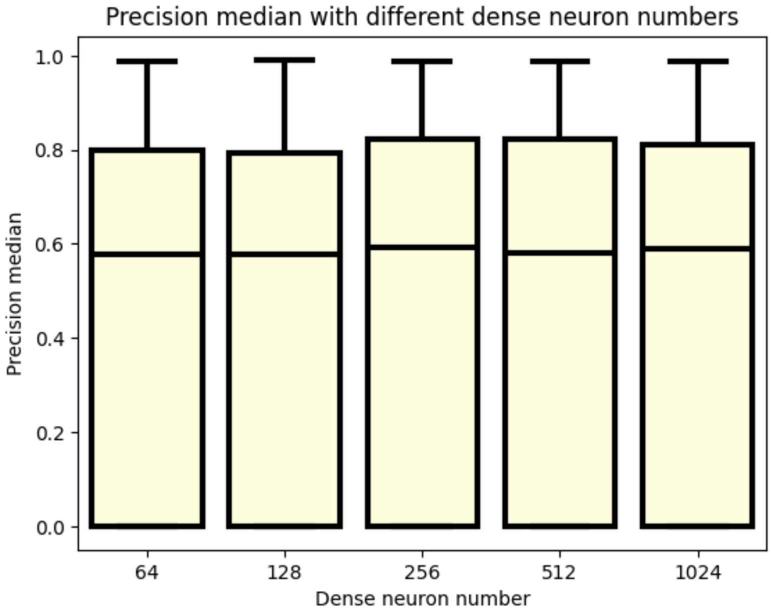

Figure S5. Precision value for different number off Dense neurons

Figure S5 depicts the examination of Precision values concerning the number of neurons in the dense layer, with values ranging from 64 to 1024. Upon analysis of the median Precision values, it is observed that this parameter has minimal to no discernible effect on model performance. The median values exhibit little variation across different neuron counts, with one notable exception at 256 neurons displaying a relatively high value. Additionally, the upper quartile values are highest for the 256-neuron configuration, albeit not significantly higher than the differences in median values. Furthermore, the maximum Precision values are consistent across all configurations, suggesting the presence of a configuration for each neuron count capable of achieving values close to 1. Thus, the number of neurons in the dense layer does not substantially impact the model's performance, as evidenced by the ability of each configuration to attain high Precision values.

2. Recall

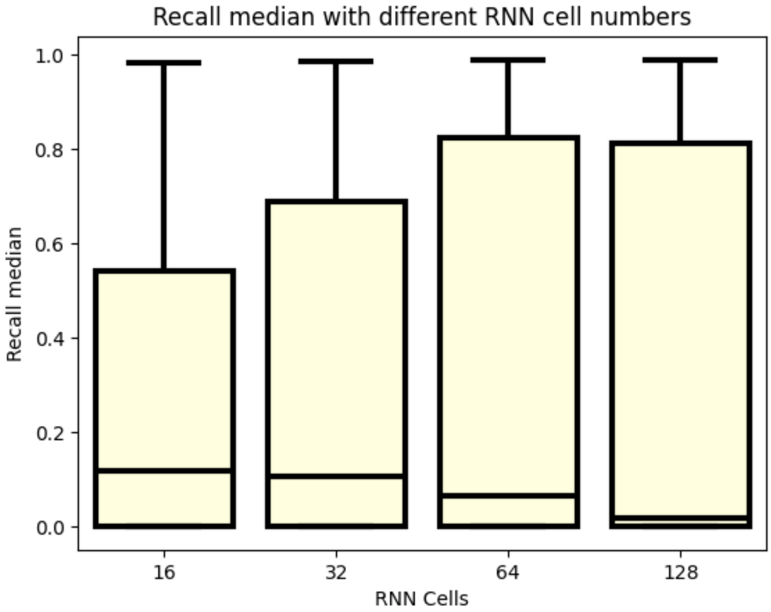

Figure S6. Recall value for different number off RNN cell

In Figure S6, the Recall metric for the set of RNN cells is examined. Similar trends are observed as with Precision; however, there are notable differences. The median of the box plots is generally lower for Recall compared to Precision. Additionally, a significant difference is noted in the upper quartile, with the highest value observed for Recall at 64 RNN cells. Despite these differences, it is evident that models with varying numbers of RNN cells perform well, as indicated by the ability of each configuration to achieve Recall values close to 1. However, the results suggest that using more than 64 RNN cells may not yield significant improvements in model performance.

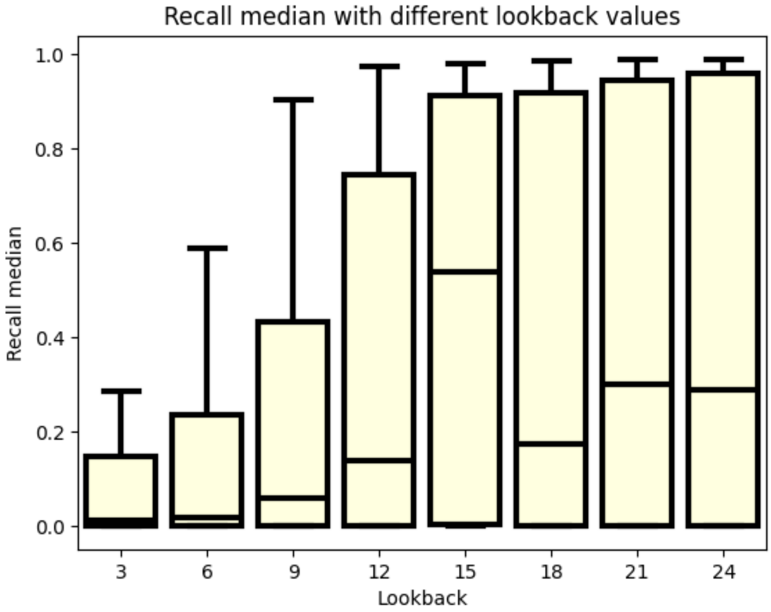

Figure S7. Recall value for different number off Look back

In Figure S7, the Recall metric is examined concerning the look-back time. A noticeably larger variability is observed compared to Precision, which is expected due to the nature

of Recall metrics where false negatives are more likely than false positives, especially with fewer positive classes. Upon analysis of the graph, it becomes apparent that smaller look-back values provide little utility. Models begin to perform well from a look-back of 9, achieving Recall values above 0.8. However, the majority of models fail to reach a value of 0.2, as indicated by the median values falling below this threshold. The highest median value is observed for a look-back of 15, but there is a significant decrease in median value for larger look-back values compared to Precision. Nevertheless, it is more beneficial to utilize a look-back of 24. This is evidenced by the highest upper quartile value and the maximum value, which approaches 1. Despite the potentially longer learning time associated with higher look-back values, the performance gains outweigh the increased learning time.

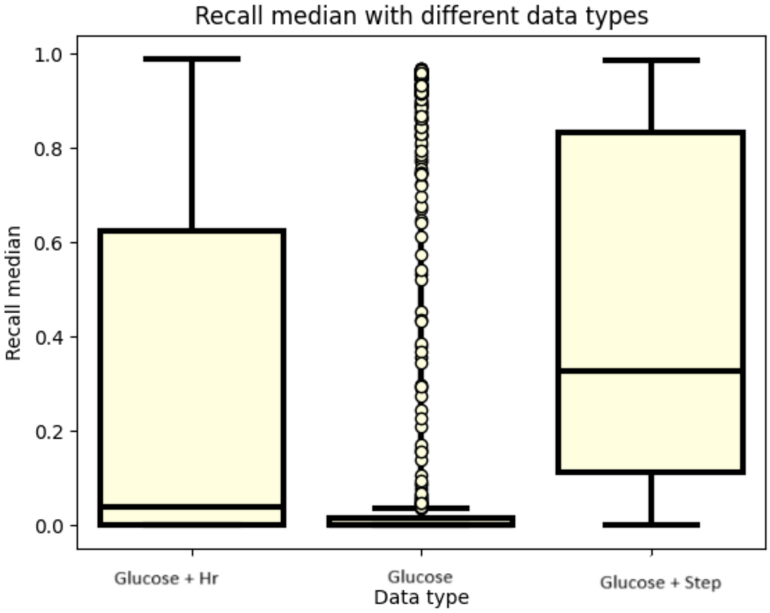

Figure S8. Recall value for different datatype

In Figure S8, the different datasets are depicted, and their respective Recall metric results are analyzed. These datasets include blood glucose level alone, blood glucose level with heart rate, and blood glucose level with step count. Analysis of the chart reveals that relying solely on blood glucose data is generally not optimal, as only outlier values approach 1 in Recall metric. However, it is noteworthy that some models can achieve high performance using this data alone. When incorporating heart rate data with blood glucose level, the median Recall value remains below 0.2, indicating suboptimal performance. Nonetheless, there are models achieving maximum Recall values close to 1, albeit without outliers. The most effective dataset configuration is observed when step count data is included with blood glucose level. The median value for this configuration is the highest among the three datasets, suggesting superior performance. Furthermore, the upper quartile value exceeds a Recall of 0.8, indicating that 25% of the models can achieve a Recall value greater than 0.8. However, it's important to note that this performance improvement is specific to this particular data configuration.

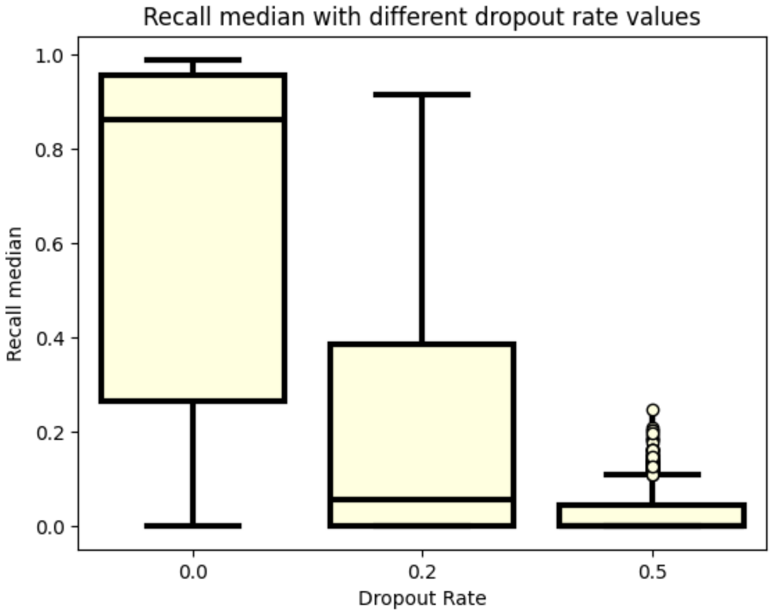

Figure S9. Recall value for different number off Drop out rate

In Figure S9, the impact of different dropout rate variants on the Recall metric is examined. The dropout rate of 0.5 exhibits the poorest performance, with no model configuration achieving a Recall value of 0 .4. Similarly, the dropout rate of 0.2 yields suboptimal results, albeit slightly better than the 0.5 dropout rate. While the maximum Recall value reaches 0.9 for the 0.2 dropout rate, it is still inferior to the performance achieved with a dropout rate of 0.0. It is evident that a dropout rate of 0.0 consistently produces the best results. The median Recall value exceeds 0.8, while both the upper quartile value and the maximum value can reach 1, indicating optimal model performance.

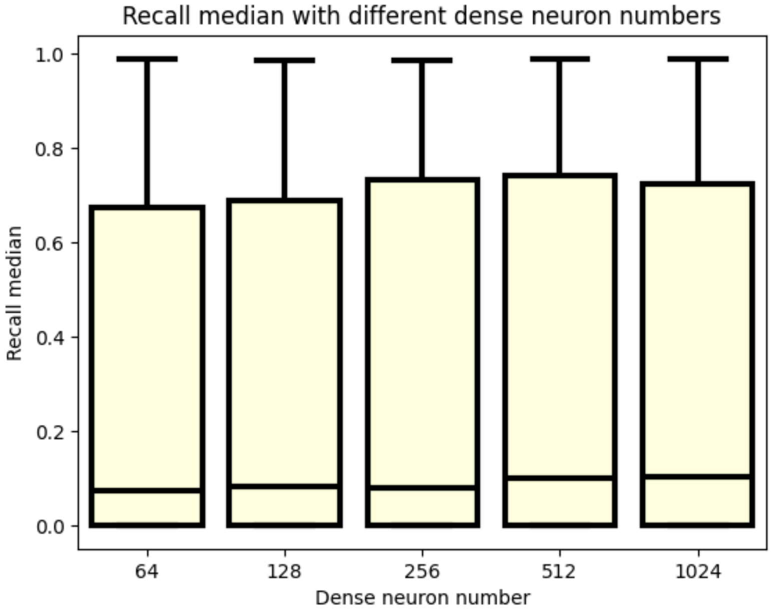

Figure S10. Recall value for different number off Dense neurons

In Figure S10, the dense layer neuron counts are plotted based on the Recall metrics. Although there is a slightly larger variability compared to the Precision case, the differences observed are not statistically significant. Other parameters have a more substantial impact on the results.It appears that utilizing 256 and 512 neuron counts in the hidden layers may

be slightly more beneficial, but these configurations do not significantly outperform others. Nevertheless, in all cases, there exists a configuration capable of achieving a maximum Recall value close to 1.

3. Accuracy

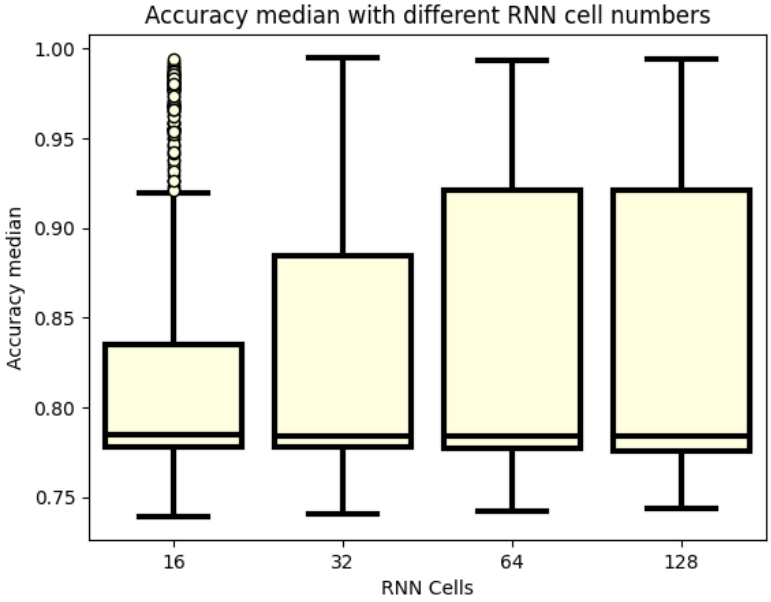

Figure S11. ACC value for different number off RNN cell

In Figure S11, precision is plotted as a function of the number of RNN cells, and their corresponding boxplots are presented. Contrary to the uniform patterns observed in Precision, Recall, and F1 score values, the accuracy metric exhibits differences across various RNN cell counts. Interestingly, models utilizing 16 cells perform notably worse than those employing larger cell counts. Notably, models using 64 and 128 cells demonstrate superior performance, with significantly larger upper quartile values exceeding 95%. Therefore, it is advisable to consider utilizing these two cell numbers to achieve higher accuracy.

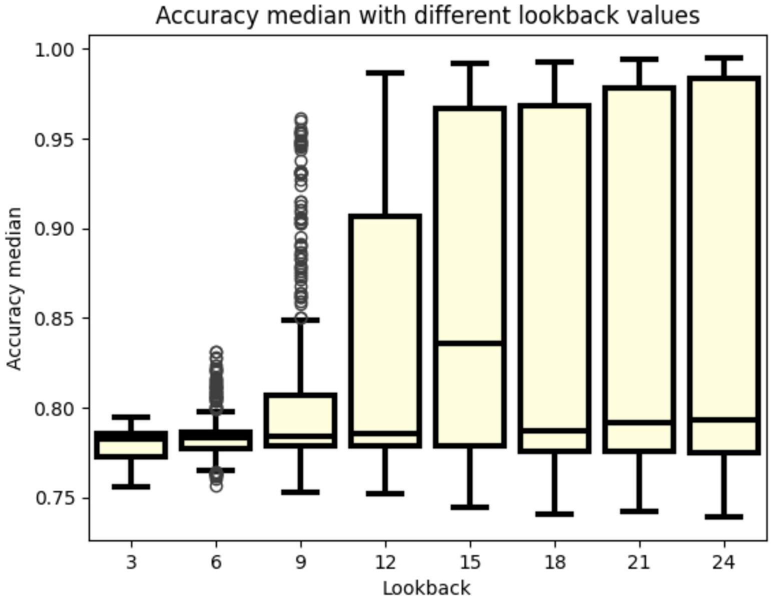

Figure S12. ACC value for different number off looc back

In Figure S12, the different look-back windows are plotted from 15 minutes to two hours. A staggered increase in performance is observed as the look-back window size increases. Outlier values demonstrate performance exceeding 80% for the 6th look-back, while for the 9th look-back, outlier values reach approximately 85% and can achieve results better than 95%. Notably, at the 12th look-back, no outliers are displayed, yet the outlier is able to reach a value above 90%. A significant improvement is observed at the 15th look-back, where the upper quartile reaches 95%, and the best median value is obtained. However, as larger look-back windows are utilized, slight improvements are observed in the upper quartile values. Furthermore, it is noteworthy that as performance improves, minimum values decrease. This suggests that larger look-back windows yield better results, but proper adjustment of other parameters is crucial. Based on accuracy, it is evident that utilizing a 24-hour look-back window is advantageous.

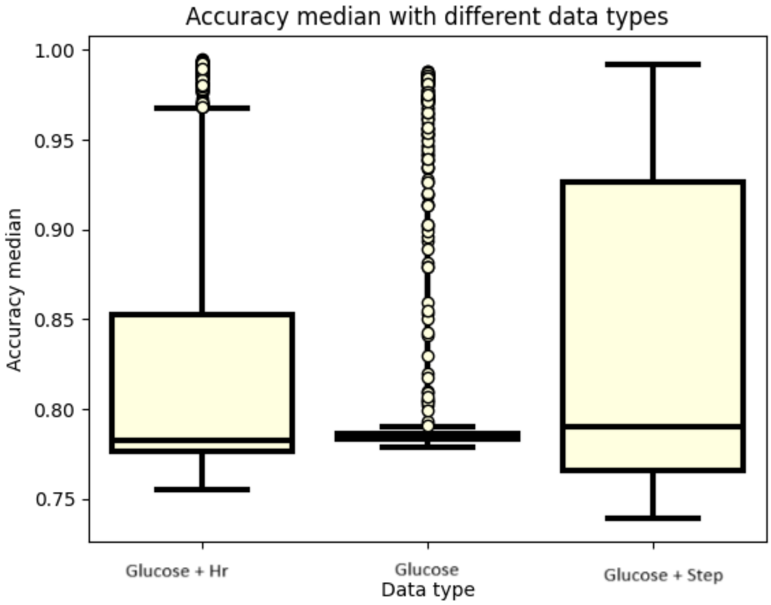

Figure S13. ACC value for different datatype

In Figure S13, accuracy is plotted for different feature datasets. It is observed that using only blood glucose levels yields the worst performance. However, similar to F1 score, Precision, and Recall, there are outlier configurations where good results can be achieved. The second-best choice for feature sets is when blood glucose levels and heart rate values are used. Unlike other metrics, outlier values are evident in this case.The best feature set choice is when both blood glucose levels and step counts are utilized. In this case, there are no outlier values, and the median accuracy is the highest. Additionally, the upper quartile exceeds 90% in this configuration alone.

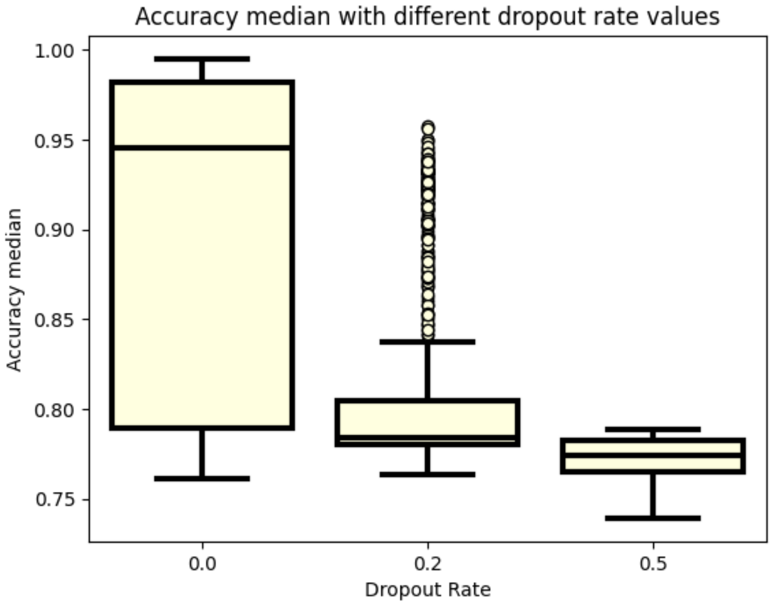

Figure S14. ACC value for different number off Drop out rate

In Figure S14, the impact of dropout rates on accuracy is examined. Similar to the cases of Precision, Recall, and F1 score, the worst performance of models is observed when dropout rates are used. Conversely, when dropout rates are not utilized, the best performance is achieved. This observation is supported by the boxplots, which demonstrate that models without dropout rates exhibit superior accuracy.Although outliers with a dropout rate of 0.2 can achieve accuracy exceeding 95%, this is not the case for a dropout rate of 0.5. Moreover, when no dropout rate is applied (dropout rate of 0.0), the median accuracy can reach almost 95%.

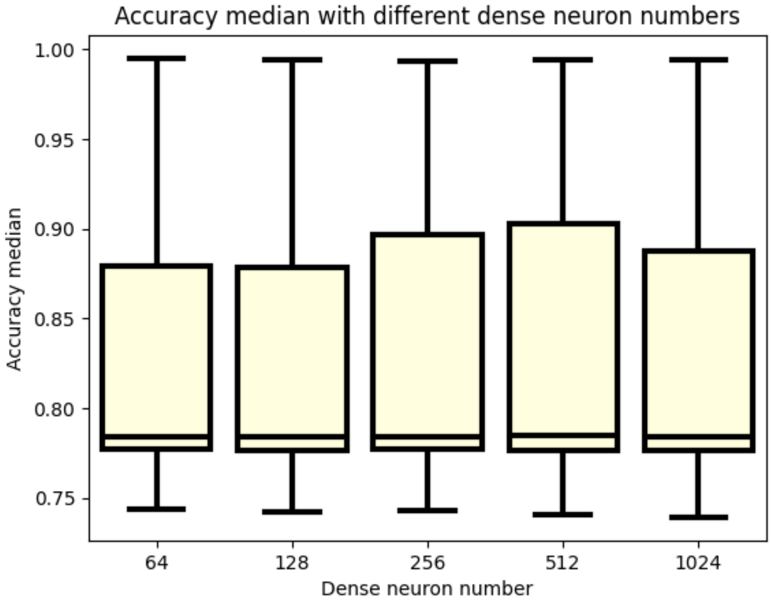

Figure S15. ACC value for different number off Dense neurons

In Figure S15, the impact of different dense layer neuron numbers on accuracy is examined. Similar to the metrics studied thus far, no deviation is observed in the performance trends. For all neuron numbers, there exists a well-performing model capable of achieving

an accuracy value of 1. Notably, the neuron numbers 256 and 512 appear to stand out, although overall, this parameter has the smallest impact on the performance of the models.

4. Analyzation of the best 30 models

In this subsection, we ranked the top 30 best F1-scoring models that we have managed to train, as the F1-score criterion provides a robust evaluation metric that balances precision and recall, ensuring that the selected models exhibit strong performance across both aspects of classification accuracy. As the table would be too large to show all metrics, we had to split it into three tables. However, the ranking of the scores based on which the top 30 models were selected is based on the median F1 score for the five test cases. This sort order has been split up to the Table S1 table with the accuracy of the AUC metric being illustrated. In the Table S2 the Precision and Recall are plotted. And in the Table 2 , the F1 score values are shown. Let's commence the analysis with Table S1, where models with

Modell	Data Type	Look back	Dropout Rate	RNN Cells	Dense Neuron Number	AUC			ACC		
						Mean	Median	STD	Mean	Median	STD
LSTM	Glucose and HR	24.0000	0.0000	32.0000	64.0000	0.9981	0.9985	0.0020	0.9930	0.9949	0.0026
LSTM	Glucose and HR	21.0000	0.0000	128.0000	128.0000	0.9994	0.9995	0.0002	0.9944	0.9942	0.0011
LSTM	Glucose and HR	24.0000	0.0000	16.0000	1024.0000	0.9994	0.9996	0.0005	0.9927	0.9940	0.0031
LSTM	Glucose and HR	24.0000	0.0000	128.0000	512.0000	0.9994	0.9993	0.0004	0.9930	0.9940	0.0023
GRU	Glucose and HR	24.0000	0.0000	128.0000	1024.0000	0.9997	0.9997	0.0002	0.9942	0.9936	0.0025
LSTM	Glucose and HR	24.0000	0.0000	64.0000	64.0000	0.9993	0.9993	0.0005	0.9925	0.9936	0.0020
GRU	Glucose and HR	24.0000	0.0000	128.0000	256.0000	0.9992	0.9993	0.0001	0.9929	0.9932	0.0021
LSTM	Glucose and HR	21.0000	0.0000	128.0000	64.0000	0.9995	0.9996	0.0003	0.9933	0.9930	0.0005
LSTM	Glucose and HR	24.0000	0.0000	128.0000	64.0000	0.9996	0.9997	0.0004	0.9935	0.9932	0.0020
GRU	Glucose and HR	24.0000	0.0000	128.0000	64.0000	0.9996	0.9997	0.0003	0.9932	0.9928	0.0016
LSTM	Glucose and HR	21.0000	0.0000	128.0000	1024.0000	0.9994	0.9993	0.0003	0.9926	0.9930	0.0024
LSTM	Glucose and HR	24.0000	0.0000	64.0000	128.0000	0.9989	0.9986	0.0006	0.9920	0.9932	0.0022
LSTM	Glucose and Stpes	24.0000	0.0000	128.0000	128.0000	0.9992	0.9991	0.0004	0.9919	0.9921	0.0020
LSTM	Glucose and HR	24.0000	0.0000	16.0000	256.0000	0.9990	0.9991	0.0007	0.9918	0.9928	0.0022
LSTM	Glucose and HR	18.0000	0.0000	128.0000	256.0000	0.9995	0.9996	0.0003	0.9929	0.9927	0.0007
LSTM	Glucose and HR	24.0000	0.0000	128.0000	128.0000	0.9997	0.9997	0.0001	0.9929	0.9932	0.0011
LSTM	Glucose and Stpes	24.0000	0.0000	128.0000	256.0000	0.9992	0.9995	0.0007	0.9917	0.9921	0.0008
GRU	Glucose and HR	24.0000	0.0000	128.0000	128.0000	0.9992	0.9992	0.0004	0.9926	0.9928	0.0013
GRU	Glucose and Stpes	24.0000	0.0000	128.0000	128.0000	0.9995	0.9994	0.0002	0.9918	0.9921	0.0027
LSTM	Glucose and HR	24.0000	0.0000	64.0000	256.0000	0.9991	0.9994	0.0007	0.9920	0.9923	0.0021
LSTM	Glucose and HR	24.0000	0.0000	128.0000	256.0000	0.9992	0.9993	0.0006	0.9927	0.9923	0.0013
LSTM	Glucose and HR	24.0000	0.0000	32.0000	256.0000	0.9989	0.9990	0.0007	0.9911	0.9928	0.0036
LSTM	Glucose and HR	24.0000	0.0000	32.0000	512.0000	0.9993	0.9991	0.0005	0.9933	0.9923	0.0020
LSTM	Glucose and Stpes	24.0000	0.0000	128.0000	64.0000	0.9991	0.9992	0.0002	0.9915	0.9912	0.0014
GRU	Glucose and HR	24.0000	0.0000	64.0000	256.0000	0.9995	0.9996	0.0002	0.9918	0.9923	0.0012
LSTM	Glucose and HR	21.0000	0.0000	128.0000	512.0000	0.9988	0.9989	0.0008	0.9906	0.9921	0.0042
GRU	Glucose and Stpes	24.0000	0.0000	128.0000	64.0000	0.9992	0.9993	0.0004	0.9912	0.9917	0.0020
GRU	Glucose and HR	24.0000	0.0000	64.0000	512.0000	0.9993	0.9992	0.0003	0.9917	0.9923	0.0018
GRU	Glucose and HR	21.0000	0.0000	64.0000	1024.0000	0.9988	0.9995	0.0015	0.9904	0.9921	0.0041
LSTM	Glucose and HR	24.0000	0.0000	32.0000	128.0000	0.9991	0.9992	0.0005	0.9923	0.9923	0.0015

Table S1. The 30 best model AUC and ACC scores

varying parameter settings are presented. This table focuses on two metrics: AUC and precision. The models are arranged in descending order based on their F1 scores. It is evident that the majority of models employ LSTM-based networks and utilize both blood glucose and heart rate data. While most models have a look-back window length of 24, two models have a length of 21. Interestingly, in the top thirty there is a model with a look back of 18. As for the drop out rate, it is quite clear that all models in the best 30 used a dropout rate of zero. Regarding RNN cells, the majority of models use either 128 or 64 cells, with some employing 32 cells, suggesting a potentially lesser impact of this parameter on model performance. However, the number of neurons in the dense layer appears to have the least influence, as the top thirty models all utilize 64, 128, 256, 512, and 1024 neurons. Examining the AUC metrics in the table reveals that all 30 models exhibit highly favorable results, with AUC values ranging from 0.998 to 0.999. The median value closely aligns with the mean value, indicating minimal variance across the models. This is further supported by the standard deviation (STD) column, where the maximum standard deviation point for the best model is 0.002. Moving on to the accuracy column, the mean, median, and standard deviation are plotted similarly. Interestingly, although the average accuracy for the second model is better than the first, the median accuracy for the first model surpasses the second. Notably, the first model, considered the best, exhibits a much higher standard

deviation, as indicated by the STD column, where the second model configuration has half the value of the first. However, despite this variance, minimal variation is observed between the best models. It can be hypothesized that the first model's superiority may stem from its ability to better learn the data in a two-training case due to its smaller size. However, generalization of this observation may not be warranted.

Modell	Data Type	Look back	Dropout Rate	RNN Cells	Dense Neuron Number	Precision			Recall		
						Mean	Median	STD	Mean	Median	STD
LSTM	Glucose and HR	24.0000	0.0000	32.0000	64.0000	0.9871	0.9873	0.0026	0.9816	0.9865	0.0109
LSTM	Glucose and HR	21.0000	0.0000	128.0000	128.0000	0.9913	0.9910	0.0050	0.9841	0.9853	0.0030
LSTM	Glucose and HR	24.0000	0.0000	16.0000	1024.0000	0.9821	0.9801	0.0082	0.9857	0.9814	0.0090
LSTM	Glucose and HR	24.0000	0.0000	128.0000	512.0000	0.9828	0.9868	0.0071	0.9865	0.9869	0.0058
GRU	Glucose and HR	24.0000	0.0000	128.0000	1024.0000	0.9869	0.9868	0.0068	0.9877	0.9888	0.0047
LSTM	Glucose and HR	24.0000	0.0000	64.0000	64.0000	0.9846	0.9833	0.0058	0.9825	0.9830	0.0055
GRU	Glucose and HR	24.0000	0.0000	128.0000	256.0000	0.9822	0.9797	0.0091	0.9858	0.9848	0.0042
LSTM	Glucose and HR	21.0000	0.0000	128.0000	64.0000	0.9850	0.9817	0.0073	0.9855	0.9871	0.0050
LSTM	Glucose and HR	24.0000	0.0000	128.0000	64.0000	0.9867	0.9865	0.0046	0.9844	0.9865	0.0064
GRU	Glucose and HR	24.0000	0.0000	128.0000	64.0000	0.9868	0.9878	0.0032	0.9839	0.9840	0.0043
LSTM	Glucose and HR	21.0000	0.0000	128.0000	1024.0000	0.9865	0.9892	0.0085	0.9817	0.9804	0.0048
LSTM	Glucose and HR	24.0000	0.0000	64.0000	128.0000	0.9809	0.9828	0.0077	0.9832	0.9845	0.0049
LSTM	Glucose and Stpes	24.0000	0.0000	128.0000	128.0000	0.9865	0.9860	0.0041	0.9814	0.9815	0.0052
LSTM	Glucose and HR	24.0000	0.0000	16.0000	256.0000	0.9828	0.9818	0.0061	0.9813	0.9798	0.0076
LSTM	Glucose and HR	18.0000	0.0000	128.0000	256.0000	0.9867	0.9858	0.0039	0.9820	0.9806	0.0050
LSTM	Glucose and HR	24.0000	0.0000	128.0000	128.0000	0.9843	0.9850	0.0054	0.9837	0.9817	0.0057
LSTM	Glucose and Stpes	24.0000	0.0000	128.0000	256.0000	0.9839	0.9833	0.0045	0.9831	0.9834	0.0053
GRU	Glucose and HR	24.0000	0.0000	128.0000	128.0000	0.9803	0.9813	0.0042	0.9873	0.9868	0.0032
GRU	Glucose and Stpes	24.0000	0.0000	128.0000	128.0000	0.9848	0.9851	0.0034	0.9824	0.9814	0.0110
LSTM	Glucose and HR	24.0000	0.0000	64.0000	256.0000	0.9871	0.9868	0.0076	0.9784	0.9778	0.0032
LSTM	Glucose and HR	24.0000	0.0000	128.0000	256.0000	0.9847	0.9854	0.0062	0.9832	0.9849	0.0081
LSTM	Glucose and HR	24.0000	0.0000	32.0000	256.0000	0.9812	0.9807	0.0091	0.9788	0.9790	0.0092
LSTM	Glucose and HR	24.0000	0.0000	32.0000	512.0000	0.9872	0.9852	0.0043	0.9830	0.9816	0.0069
LSTM	Glucose and Stpes	24.0000	0.0000	128.0000	64.0000	0.9852	0.9856	0.0076	0.9815	0.9806	0.0070
GRU	Glucose and HR	24.0000	0.0000	64.0000	256.0000	0.9860	0.9853	0.0031	0.9788	0.9761	0.0051
LSTM	Glucose and HR	21.0000	0.0000	128.0000	512.0000	0.9800	0.9866	0.0109	0.9773	0.9772	0.0100
GRU	Glucose and Stpes	24.0000	0.0000	128.0000	64.0000	0.9843	0.9832	0.0034	0.9802	0.9832	0.0056
GRU	Glucose and HR	24.0000	0.0000	64.0000	512.0000	0.9770	0.9776	0.0058	0.9861	0.9887	0.0061
GRU	Glucose and HR	21.0000	0.0000	64.0000	1024.0000	0.9809	0.9823	0.0105	0.9767	0.9754	0.0109
LSTM	Glucose and HR	24.0000	0.0000	32.0000	128.0000	0.9874	0.9869	0.0015	0.9779	0.9793	0.0087

Table S2. The 30 best model Precision and Recall scores

Next is the Table S2, where the Precision and Recall metrics are presented in a similar manner. Upon examination of the table, it is evident that the models yield exceptionally high results. Notably, the weakest Precision value among the top thirty models is 0.97, with an average value close to 0.98. This trend is mirrored in the Recall metric, where similarly strong results are observed. However, it is worth noting that these metrics exhibit a larger variance compared to Precision and AUC. Nevertheless, achieving a Precision value of 0.99, as demonstrated by our second-best model, is a noteworthy accomplishment. Interestingly, the variance is higher for the best model compared to the second best, indicating greater volatility in the former.
